# Malaria Infections and Placental Blood Flow: A Doppler Ultrasound Study From a Preconception Cohort in Benin

**DOI:** 10.1093/ofid/ofad376

**Published:** 2023-08-10

**Authors:** Aude Mondeilh, Emmanuel Yovo, Manfred Accrombessi, Cornelia Hounkonnou, Gino Agbota, William Atade, Olaiitan T Ladikpo, Murielle Mehoba, Auguste Degbe, Bertin Vianou, Dariou Sossou, Nicaise Tuikue Ndam, Achille Massougbodji, Rose McGready, Nadine Fievet, Marcus J Rijken, Gilles Cottrell, Valérie Briand

**Affiliations:** Research Institute for Sustainable Development (IRD) EMR 271, Bordeaux Population Health Centre, National Institute for Health and Medical Research (INSERM) UMR 1219, University of Bordeaux, Bordeaux, France; Institut de Recherche Clinique du Bénin (IRCB), Abomey-Calavi, Benin; Montpellier Interdisciplinary Center on Sustainable Agri-food Systems (MoISA), Université de Montpellier, Montpellier, France; Institut de Recherche Clinique du Bénin (IRCB), Abomey-Calavi, Benin; Faculty of Infectious and Tropical Diseases, Disease Control Department, London School of Hygiene and Tropical Medicine London, United Kingdom; Centre d'investigation clinique, module épidémiologie clinique (CIC-EC 1425), Université Paris Cité and Université Sorbonne Paris Nord, Paris, France; Département d’Épidémiologie, Biostatistique et Recherche Clinique, AP-HP, Hôpital Bichat Paris, France; Institut de Recherche Clinique du Bénin (IRCB), Abomey-Calavi, Benin; Institut de Recherche Clinique du Bénin (IRCB), Abomey-Calavi, Benin; Institut de Recherche Clinique du Bénin (IRCB), Abomey-Calavi, Benin; Institut de Recherche Clinique du Bénin (IRCB), Abomey-Calavi, Benin; Institut de Recherche Clinique du Bénin (IRCB), Abomey-Calavi, Benin; Institut de Recherche Clinique du Bénin (IRCB), Abomey-Calavi, Benin; Institut de Recherche Clinique du Bénin (IRCB), Abomey-Calavi, Benin; Research Institute for Sustainable Development (IRD), UMR 261 MERIT, Université Paris Cité, Paris, France; Institut de Recherche Clinique du Bénin (IRCB), Abomey-Calavi, Benin; Shoklo Malaria Research Unit, Mahidol Oxford Tropical Medicine Research Unit, Faculty of Tropical Medicine, Mahidol University, Mae Sot, Thailand; Centre for Tropical Medicine and Global Health, Nuffield Department of Medicine, University of Oxford, Oxford, United Kingdom; Research Institute for Sustainable Development (IRD), UMR 261 MERIT, Université Paris Cité, Paris, France; Julius Global Health, Julius Center for Health Sciences and Primary Care, University Medical Center Utrecht, Utrecht University, Utrecht, The Netherlands; Department of Obstetrics and Gynecology, University Medical Center Utrecht, Utrecht University, Utrecht, The Netherlands; Research Institute for Sustainable Development (IRD), UMR 261 MERIT, Université Paris Cité, Paris, France; Research Institute for Sustainable Development (IRD) EMR 271, Bordeaux Population Health Centre, National Institute for Health and Medical Research (INSERM) UMR 1219, University of Bordeaux, Bordeaux, France

**Keywords:** Africa, Doppler measurements, malaria in pregnancy, umbilical artery, uterine artery

## Abstract

**Background:**

Malaria in pregnancy (MiP) has been associated with fetal growth restriction, the underlying pathogenic mechanisms of which remain poorly understood. Malaria in pregnancy is suspected to induce abnormalities in placental vascularization, leading to impaired placental development. Our study evaluated MIP's effect on uterine artery (UtA) and umbilical artery (UA) blood flow.

**Methods:**

The analysis included 253 Beninese women followed throughout pregnancy and screened monthly for submicroscopic and microscopic malaria. Uterine artery Doppler measurement was performed once between 21 and 25 weeks’ gestation (wg), and UA Doppler measurement was performed 1–3 times from 28 wg. Linear and logistic regression models were used to assess the effect of malaria infections on UtA Doppler indicators (pulsatility index and presence of a notch), whereas a logistic mixed model was used to assess the association between malaria infections and abnormal UA Doppler (defined as Z-score ≥2 standard deviation or absent/reversed UA end-diastolic flow).

**Results:**

Primigravidae represented 7.5% of the study population; 42.3% of women had at least 1 microscopic infection during pregnancy, and 29.6% had at least 1 submicroscopic infection (and no microscopic infection). Both microscopic and submicroscopic infections before Doppler measurement were associated with the presence of a notch (adjusted odds ratio [aOR] 4.5, 95% confidence interval [CI] = 1.2–16.3 and aOR 3.3, 95% CI = .9–11.9, respectively). No associations were found between malaria before the Doppler measurement and abnormal UA Doppler.

**Conclusions:**

Malaria infections in the first half of pregnancy impair placental blood flow. This highlights the need to prevent malaria from the very beginning of pregnancy.

Pregnant women are a population that is particularly at risk of malaria [1]. In sub-Saharan Africa (SSA), the main deleterious effect of malaria for the fetus is an increased risk of low birth weight and small for gestational age (SGA), due to fetal growth restriction (FGR), prematurity, or a combination of both [2]. Although the impact of malaria in pregnancy (MiP) on poor birth outcomes has been well documented, pathogenesis of MiP-associated FGR remains poorly understood.

Malaria in pregnancy is characterized by both the sequestration and accumulation of infected erythrocytes in the intervillous blood space of the placenta, a key organ for fetal development [3]. Different pathological pathways have been considered to explain MiP-associated FGR. In particular, it has been suggested that placental malaria infections may alter placental development, thereby inducing abnormalities in placental vascularization [4–6]. On the maternal side, increased uterine artery (UtA) resistance in the first half of pregnancy indicates an impaired trophoblast invasion, which is a crucial stage in placental development [7, 8]. On the fetal side, increased umbilical artery (UA) resistance at the end of pregnancy reflects increased placental vascular resistance. Both maternal and fetal vascular abnormalities result in impaired placental functions, in turn, affecting fetal development [9, 10]. Studies on MiP using Doppler ultrasound—a real-time method to investigate placental blood flow characteristics—have been limited so far because ultrasound is still rarely used in SSA [11–15]. The “REtard de Croissance Intra-uterin et PALudisme” (RECIPAL) study, based on a cohort of women living in Benin and followed from preconception to delivery, assessed the impact of MiP on uterine and umbilical artery resistance indices, as measured by Doppler ultrasound.

## METHODS

### Patient Consent Statement

This study was approved by the Ethics Committee of the Institut des Sciences Biomédicales Appliquées as well as the Ministry of Health in Benin. Written informed consent was obtained from all participants after an explanation of the study in the local language. The project funded treatment for malaria and other acute diseases during pregnancy.

### Study Design

RECIPAL is a preconception cohort study conducted between June 2014 and August 2017 in southern Benin, where *Plasmodium falciparum* is the most common malaria-causing parasite [16]. Women of reproductive age were recruited at community level and followed monthly at home for a maximum of 24 months until they became pregnant (primary cohort). Once the pregnancy was confirmed, they were followed monthly in the maternity clinic until delivery (secondary cohort) (Supplementary Figure 1). The complete study protocol has been detailed elsewhere [17].

### Data Collection

At inclusion in the primary cohort, sociodemographic and anthropometric data and reproductive history were collected. At each monthly visit, the date of the last menstrual period (LMP) was recorded, and a urinary pregnancy test was performed.

During gestational follow up, anthropometric, clinical, and obstetrical data were collected at each monthly antenatal care (ANC) visit. Women were also screened for malaria using both microscopy (thick blood smear [TBS]) and polymerase chain reaction [PCR]). In addition, any time during pregnancy, a rapid diagnostic test (RDT) (*P falciparum* + standard deviation [SD] Bioline Malaria-Ag-Pf/Pan rapid test; IDA Foundation, Amsterdam, Netherlands; Biosynex, Strasbourg, France) and a TBS were performed in case of fever or other signs pointing to malaria. Because PCR results were only available at the end of the study, only women infected with malaria based on TBS and/or RDT were treated during pregnancy according to national guidelines (ie, quinine in the first trimester and artemisinin-combination therapy in the second and third trimesters).

Ultrasound scans (USs) were performed using a portable ultrasound scanner (5–2 MHz C60 abdominal probe; Sonosite M-TURBO; Bothell, Washington). To date the pregnancy, the first US was performed between 9 and 14 weeks of gestation (wg). Crown-rump length measurement was used to estimate gestational age (GA) at the first US performed between 9 and 13 wg according to LMP. At the end, GA estimation was based either on LMP if the difference between the 2 measurements (LMP/US) was less than 7 days or on US if the difference was >7 days [18]. Additional USs were performed to assess fetal biometry every 6 weeks from 15 wg.

In particular, a color-pulsed Doppler ultrasound was used (1) to interrogate flow velocity waveform in the left and right UtA once between 21 and 25 wg and (2) in the UA a maximum 3 times from 28 wg. The right and left UtA were identified at the crossover with the external iliac arteries. When 4–6 similar consecutive waveforms were obtained, UtA pulsatility index (UtA-PI) was measured, and the presence of a notch was determined. The UA Doppler flow was assessed on a free loop of cord in the absence of fetal breathing and body movement. When 4–6 uniform consecutive waveforms were obtained, umbilical artery PI (UA-PI) was measured, and UA end-diastolic flow was recorded as present, absent, or reversed. Doppler USs were performed successively by 4 sonographers during the study period. Quality control for all UtA Dopplers and for a random subsample of UA Dopplers was performed a posteriori by an experienced obstetrician-gynecologist.

### Laboratory Procedures

For TBS analysis, the Lambarene technique was used to quantify parasitemia with a detection threshold estimated at 5 parasites/μL [19]. For real-time quantitative PCR, 18S ribosomal deoxyribonucleic acid (DNA) was targeted [20, 21]. A negative control with no DNA template was run in all reactions.

### Statistical Analysis

Our main objective was to evaluate the impact of MiP on UtA and UA Doppler measurements. Our study population included the subsample of women with a viable and single pregnancy, who had at least 1 UtA Doppler and/or UA Doppler for which quality was checked and with available TBS and PCR results before each considered Doppler measurement.

For UtA Doppler analyses, the outcomes were as follows: UtA-PI (highest measurement between left and right UtA) and notch (presence on left or right UtA vs absence). In fact, with increasing GA, a high UtA-PI and/or the presence of a notch reflect high vascular resistance [22]. For UA Doppler analyses, the outcome was a binary variable (abnormal UA Doppler vs normal) built in 2 steps. First, UA-PI was transformed into a Z-score according to INTERGROWTH-21st charts [23]. Then, “abnormal UA Doppler” was defined as follows: Z-score ≥2 SD (equivalent to UA-PI ≥98th percentile) or absent/reversed UA end-diastolic flow (ultimate stage of an abnormal UA Doppler) [24].

Our main exposure variable was the time-dependent malaria status, categorized into 3 classes and created as follows: (1) first, at each scheduled and unscheduled ANC visit, malaria status was defined as “negative” if all tests (TBS, PCR, and RDT when applicable) were negative; malaria status was defined aas “submicroscopic” if PCR test was positive and TBS and/or RDT tests were negative; and malaria status was defined as “microscopic” if TBS and/or RDT tests were positive; (2) then, we created a summary variable based on the woman's malaria status from the beginning of pregnancy up to the day of Doppler measurement. Thus, for a given Doppler measurement, malaria status was summarized as follows: no malarial infection detected before the Doppler measurement; at least 1 submicroscopic infection, but no microscopic infection detected before the Doppler measurement; or at least 1 microscopic infection detected before the Doppler measurement, independently of the occurrence of a submicroscopic infection. Similar variables summarizing the infection status at the first (ie, before 15 wg) and the second trimester (15–28 wg) of pregnancy were also created using the same procedure. These variables were used for secondary analysis on UtA Doppler measurement.

First, we described the women's general and malaria characteristics along with the UtA and UA Doppler measurements’ timing and values. The crude association between UtA notching and abnormal UA Doppler with poor birth outcomes was evaluated. Poor birth outcomes were defined as low birth weight (birth weight <2500 grams), preterm birth (GA <37 wg), and SGA (defined as birth weight below the 10th percentile for a given GA according to INTERGROWTH-21st's charts) [18].

Next, we assessed the association between malaria status before UtA Doppler measurement and UtA Doppler indicators using linear regression (for UtA-PI) and logistic regression (for notch) models. Similar models were used to assess the effect of malaria in the first and second trimesters on UtA-PI and notch specifically. A logistic mixed model with a random intercept on the woman's identification was used to assess the association between MiP and “abnormal UA Doppler,” to account for possible correlation between repeated UA Doppler measurements on a single woman.

Potential confounders were identified a priori using directed acyclic graphs (DAGs). Based on DAGs, UtA and UA analyses were adjusted for maternal age and GA (both continuous variables), along with gravidity (primigravidae/secondigravidae vs multigravidae), ethnicity (Toffin vs others), and socioeconomic status (SES). The SES was based on a score calculated according to the woman's occupation and material assets and then categorized by tertiles [25].

Coefficients with a *P* value less than .05 were considered as statistically significant. All statistical analyses were performed using RStudio software version 1.3.1073.

## RESULTS

### Study Population

**Figure 1.**
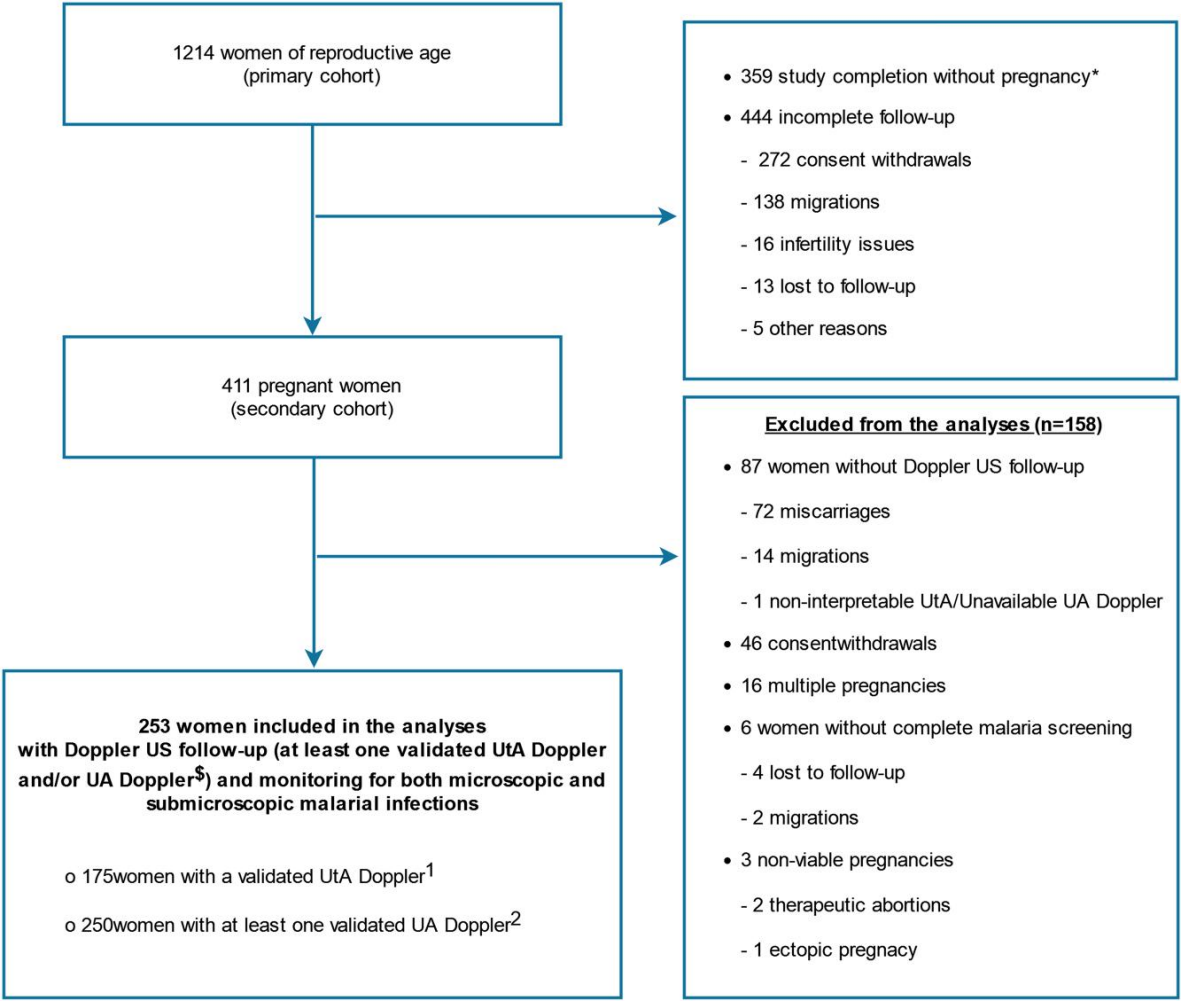
Flowchart diagram of the study population, REtard de Croissance Intra-uterin et PALudisme (RECIPAL) cohort, southern Benin, 2014–2017. *Study completion: 24-month follow up without pregnancy from enrollment. ^$^All uterine artery (UtA) Dopplers and a random subsample of umbilical artery (UA) Dopplers were validated a posteriori by an experienced obstetrician-gynecologist. ^1^For UtA analysis, 78 women without UtA Doppler (69 noninterpretable UtA Doppler and 9 with UA Doppler only) were excluded.^2^ For UA analysis, 3 women without UA Doppler (3 with validated UtA Doppler only) were excluded.

Out of the 1214 women of reproductive age included in the primary cohort, 411 (33.8%) became pregnant after 30 months of follow up (Figure 1). A total of 253 women (61.6%) with a single and viable pregnancy were selected for this analysis, 175 of whom had a validated UtA Doppler and 250 had at least 1 validated UA Doppler. All of them were monitored for both microscopic and submicroscopic malarial infections. Overall, 158 of 411 were excluded from the analyses; the main reasons for exclusion were miscarriage and consent withdrawal (Figure 1). Women included in (n = 253) and excluded from (n = 158) the analysis did not differ significantly in their general characteristics (Supplementary Table 1).

Quality control of Doppler-USs led to the exclusion of 69 women with uninterpretable UtA Doppler. Only 4 UA Doppler images were considered uninterpretable, which did not lead to exclusion of the women concerned, who had 1 or 2 other interpretable UA Doppler measurements. We did not find any differences between the 69 women with uninterpretable UtA Doppler and the 175 women included in the UtA analyses (Supplementary Table 2).

### Women's Characteristics Before and During Pregnancy

At inclusion, women were on average 27 years old (Table 1), few women (7.5%) were primigravidae, the majority (71.5%) were illiterate, and more than half (57.7%) were anemic. Women had a mean of 7.7 ANC visits, and the average GA at the first ANC visit was 7 wg. The proportion of women who had at least 1 microscopic infection during pregnancy was 42.3%, whereas those who had at least 1 submicroscopic infection (and no microscopic infection) were 29.6%. In the first trimester, these proportions were 19.8% and 28.2%, respectively.

**Table 1.** General Characteristics of the 253 Pregnant Women Included in the Analyses, RECIPAL Cohort, Southern Benin, 2014–2017

Characteristics	Total	N (%) or Mean (SD)
Maternal age (years)	253	26.7 (5.0)
Gravidity	253	…
Primigravidae	…	19 (7.5)
Secondigravidae	…	32 (12.6)
Multigravidae	…	202 (79.8)
Education level (illiterate vs literate)	253	181 (71.5)
Prepregnancy body mass index (kg/m^2^)	253	…
<18.5	…	22 (8.7)
18.5–24	…	171 (67.6)
≥25	…	60 (23.7)
Socioeconomic Status^a^	253	…
Low tertile	…	87 (34.4)
Intermediate tertile	…	98 (38.7)
High tertile	…	68 (26.9)
HIV status (positive vs negative)	246	4 (1.6)
Anemia before conception (≤12 g/dL)^b^	253	146 (57.7)
Ethnic group (Toffin vs others^c^)	253	187 (73.9)
Median (range) number of ANC visits during pregnancy^d^	253	8 (4–9)
GA at the first ANC visit (weeks of gestation)^e^	253	7 (2.6)
Gestational hypertension (detected vs not)	253	7 (2.8)
SGA^f^ (yes vs no)	248	51 (20.6)
Low birthweight (<2500 grams) (yes vs no)	250	22 (8.8)
Preterm birth (yes vs no)	252	21 (8.3)
Stillbirth (yes vs no)	251	2 (0.8)
Number of malaria screenings during pregnancy	253	8.1 (1.3)
Malaria status during pregnancy	253	…
No malaria infection	…	71 (28.1)
At least 1 submicroscopic infection and no microscopic infection^g^	…	75 (29.6)
At least 1 microscopic infection	…	107 (42.3)
Malaria status in the first trimester of pregnancy	248	…
No malaria infection	…	129 (52.0)
At least 1 submicroscopic infection and no microscopic infection^g^	…	70 (28.2)
At least 1 microscopic infection	…	49 (19.8)

Abbreviations: ANC, antenatal care; GA, gestational age; HIV, human immunodeficiency virus; RECIPAL, REtard de Croissance Intra-uterin et PALudisme; SD, standard deviation; SGA, small for gestational age.

a Socioeconomic status: synthetic score combining occupation and ownership of assets, where was then categorized according to the tertiles (24).

b Anemia detected at recruitment in the preconception period, in median 12.3 months before pregnancy (17).

c Other ethnic groups: Fon, Aïzo, Yoruba, Adja, Goun, Ahoussa, Cotafon, Mahi, and Sahoue.

d Including scheduled and unscheduled ANC visits.

e Gestational age based on the first ultrasound scan or the date of the last menstrual period.

f Defined as birth weight <10th percentile for a given GA according to INTERGROWTH-21st charts (17).

g Submicroscopic infections are defined by polymerase chain reaction-positive but thick blood smear-negative infections.

### Doppler Measurements and Association With Poor Birth Outcomes

The mean GA at the time of UtA Doppler measurement was 22.6 wg (Table 2). The mean UtA-PI was 2.1, and 14.3% of women had a notch. Analyses using UA Doppler measurements were performed on 250 women: 250 women had at least 1 measurement, 208 women had at least 2 measurements, and 36 women had at least 3 measurements. The mean GA at the first UA Doppler was 29.1 wg, 34.4 wg at the second Doppler, and 37.7 wg at the third Doppler. Of the 494 UA Dopplers, 39 (7.9%) were considered abnormal, 14 of which were due to the absence or reversal of UA end-diastolic flow; 29 women (11.6%) had 1 abnormal UA Doppler, and 5 women (2%) had 2.

**Table 2.** Doppler Measurements Among the 253 Pregnant Women Included in the Analyses, RECIPAL Cohort, Southern Benin, 2014–2017

Characteristics	Total	N (%) or Mean (SD)
UtA Doppler		
Women with a validated UtA^a^	253	175 (69.2)
GA at the time of UtA Doppler (weeks of gestation)^b^	175	22.6 (1.2)
UtA-PI^c^	175	2.1 (0.5)
Presence of a notch^d^	175	25 (14.3)
GA at the time of UtA notching	25	22.3 (1.3)
UA Doppler		
Women with a validated UA^a^		
At least 1	253	250 (98.8)
At least 2	253	208 (82.2)
At least 3	253	36 (14.2)
GA at the time of UA Doppler (weeks of gestation)		
First measurement	250	29.1 (2.2)
Second measurement	208	34.4 (1.4)
Third measurement	36	37.7 (1.7)
Z-score of UA-PI^e^		
First measurement	250	0.14 (1.0)
Second measurement	208	0.09 (1.3)
Third measurement	36	0.45 (1.1)
Absent or reversed UA end-diastolic flow	494	14 (2.8)
Abnormal UA Doppler^f^	494	39 (7.9)
First measurement	250	12 (4.8)
Second measurement	208	20 (9.6)
Third measurement	36	7 (19.4)
Women with an abnormal UA Doppler		
1 episode	250	29 (11.6)
2 episodes	250	5 (2.0)

Abbreviations: GA, gestational age; PI, pulsatility index; RECIPAL, REtard de Croissance Intra-uterin et PALudisme; SD, standard deviation; UA, umbilical artery; UtA, uterine artery.

a UtA and UA Doppler validation made a posteriori by an experienced obstetrician-gynecologist.

b Gestational age based on the first ultrasound scan or the date of the last menstrual period.

c Highest pulsatility index between right and left uterine arteries.

d Presence on the right or left uterine artery.

e Z-score of pulsatility index according to INTERGROWTH-21st charts (22).

f Abnormal UA Doppler defined as follows: Z-score of UA-PI ≥2 SD according to INTERGROWTH21st charts (22) or absent/reversed UA end-diastolic flow.

The associations of UtA notching and abnormal UA Doppler with poor birth outcomes are presented in Supplementary Tables 3 and 4. There was a significantly higher proportion of SGA in women who had at least 1 abnormal UA Doppler (23.5% vs 10.7%, *P* = .02).

### Effect of Malaria in Pregnancy on Uterine Artery Doppler Measurements

We did not find any statistically significant difference in mean UtA-PI between women infected with microscopic or submicroscopic malaria infections before Doppler measurement compared to uninfected women, after adjustment for maternal age, GA at the time of the UtA-PI measurement, gravidity, ethnicity, and SES (Table 3). Similarly, when defining malaria exposure as infections that occurred in the first trimester only, we found no evidence of any difference in mean UtA-PI between infected and uninfected women (Table 3).

**Table 3.** Relation Between the UtA-PI and Malaria According to the Timing and Type (Microscopic vs Submicroscopic) of Infections During Pregnancy (n = 175): Multivariate Linear Regression (RECIPAL Cohort, Southern Benin, 2014–2017)

		Univariate Analysis		Multivariate Analysis^a^	
Exposure	n (%)	Estimated Mean Difference of UtA-PI (95% CI)	*P* ^b^	Estimated Mean Difference of UtA-PI (95% CI)	*P* ^b^
Malaria Before or at the Time of UtA Doppler	…	…	.46	…	.57
No infection	70 (40)	…	…	…	…
Submicroscopic infection(s) only^c^	56 (32)	.04 (−0.12 to 0.20)	…	0.03 (−0.14 to 0.20)	…
At least 1 microscopic infection^d^	49 (28)	0.11 (−0.06 to 0.28)	…	0.09 (−0.08 to 0.27)	…
Malaria in the 1st Trimester of Pregnancy	…	…	.95	…	.60
No infection	94 (53.7)	…	…	…	…
Submicroscopic infection(s) only^c^	50 (28.6)	0.01 (−0.15 to 0.16)	…	−0.03 (−0.20 to 0.15)	…
At least 1 microscopic infection^d^	31 (17.7)	−0.03 (−0.22 to 0.16)	…	−0.10 (−0.30 to 0.10)	…

Abbreviations: CI, confidence interval; RECIPAL, REtard de Croissance Intra-uterin et PALudisme; UtA-PI, uterine artery pulsatility index.

a Adjusted for maternal age, gestational age at time of Doppler measurement, gravidity, ethnic group, and socioeconomic status. First trimester analysis additionally adjusted for malaria infections that occurred in the second trimester before or at the time of Doppler measurement.

b Partial Fisher’s test.

c At least 1 submicroscopic infection and no microscopic infection during the period considered.

d At least 1 microscopic infection during the period considered.

In contrast, after adjustment for the same potential confounders, both microscopic and submicroscopic infections before Doppler measurement were substantially associated with the presence of a notch (adjusted odds ratio [aOR] 4.5, 95% confidence interval [CI] = 1.2–16.3 and aOR 3.3, 95% CI = .9–11.9, respectively) (Table 4). Due to the low number of both adverse events (n = 25 notches) and women with malaria infections in the first trimester, their association could not be assessed.

**Table 4.** Relation Between Uterine Artery (UtA) Notching and Malarial Infections Before or at the Time of Doppler Measurement (n = 175): Multivariate Logistic Regression (RECIPAL Cohort, Southern Benin, 2014–2017)

			Univariate Analysis		Multivariate Analysis	
Exposure	Total	Presence of a Notch^a^ n (%)	OR (95% CI)	*P* ^b^	aOR^c^ (95% CI)	*P* ^b^
Malaria Before or at the Time of UtA Doppler	…	…	…	.02	…	0.04
** **No infection	70	4 (5.7)	1	…	1	…
** **Submicroscopic infection(s) only^d^	56	10 (17.9)	3.6 (1.1–12.1)	…	3.3 (0.9–11.9)	…
** **At least 1 microscopic infection^e^	49	11 (22.4)	4.8 (1.4–16.1)	…	4.5 (1.2–16.3)	…

Abbreviations: aOR, adjusted odds ratio; CI, confidence interval; OR, odds ratio; RECIPAL, REtard de Croissance Intra-uterin et PALudisme; UtA, uterine artery.

a Presence of a notch on the right or left uterine artery.

b Likelihood ratio test.

c Adjusted for maternal age, gestational age at the time of Doppler measurement, gravidity, ethnic group and socioeconomic status.

d At least 1 submicroscopic infection and no microscopic infection before Doppler measurement.

e At least 1 microscopic infection before Doppler measurement.

### Effect of Malaria in Pregnancy on Umbilical Artery Doppler Measurements

We did not find any association between microscopic and submicroscopic malaria infections before the Doppler measurement and abnormal UA Doppler (Table 5). The multivariate model was adjusted for gravidity, maternal age, and GA only due to convergence issues.

**Table 5.** Relation Between Abnormal Umbilical Artery (UA) Doppler and Malarial Infections Before or at the Time of Doppler Measurement (250 Women and 494 UA Doppler Measurements): Multivariate Logistic Mixed Regression With a Random Intercept (RECIPAL Cohort, Southern Benin, 2014–2017)

			Univariate Analysis		Multivariate Analysis^a^	
Exposition	Total	Abnormal UA Doppler^b^ n (%)	OR (95% CI)	*P* ^c^	aOR (95% CI)	*P* ^c^
Malaria before or at the time of UA Doppler^d^	…	…	…	0.81	…	0.98
No infection	155	14 (9.0)	1	…	1	…
Submicroscopic infection(s) only^e^	157	10 (6.4)	1.4 (0.2–10.1)	…	1.2 (0.1–9.8)	…
At least 1 microscopic infection^f^	182	15 (8.2)	1.9 (0.3–13.0)	…	1.2 (0.2–9.5)	…

Abbreviations: aOR, adjusted odds ratio; CI, confidence interval; OR, odds ratio; RECIPAL, REtard de Croissance Intra-uterin et PALudisme; UA, umbilical artery.

a Adjusted for maternal age, gestational age at time of Doppler measurement, and gravidity.

b Abnormal UA defined as follows: Z-score of UA-PI ≥2 standard deviation according to INTERGROWTH21st charts (22) or absent/reversed UA end-diastolic flow.

c Likelihood ratio test.

d First UA Doppler performed in average at 29.1 weeks of gestation (wg), 34.4 wg for the second UA Doppler, and 37.7 wg for the third UA Doppler.

e At least 1 submicroscopic infection and no microscopic infection before Doppler measurement.

f At least 1 microscopic infection before Doppler measurement.

## DISCUSSION

Using malaria and Doppler US data collected from the first trimester of pregnancy, we showed an association between microscopic malaria infections and the presence of a notch on UtA at 22–23 wg, suggesting an adverse effect of malaria on placental vascularization. Submicroscopic malarial infections were also associated with UtA notching. However, we did not find any evidence supporting an association between MiP and abnormal UA Doppler at the end of pregnancy.

The original study design of the RECIPAL cohort allowed us to identify pregnant women at an early stage of their pregnancy. In addition, we were able to monitor malarial infections, both microscopic and submicroscopic, from the first trimester of pregnancy as well as fetal parameters and Doppler measurements longitudinally, which has rarely been done in SSA studies. This allowed GA to be accurately estimated using US, which was particularly important because vascular resistance indices decrease with advancing GA in normal pregnancies.

We found high values of UtA-PI compared to those generally reported in the literature in Caucasian women (reference for 95th percentile of UtA-PI is approximately 1.41 at 23 wg) [26, 27]. Indeed, in our study, the mean value of UtA-PI measured at a mean of 22.6 wg was 2.1. We are quite confident in the high recorded values because they were found by the 4 sonographers, and the quality of UtA Doppler was checked a posteriori by an experienced obstetrician-gynecologist. In addition, we found positive Z-scores whichever the first, second or third UA Doppler, meaning that UA-PIs were globally higher than international references values established by the INTERGROWTH-21st initiative [23]. Indeed, higher UA-PIs were expected because our study population was at higher risk for FGR compared to women selected for INTERGROWTH-21st because of high rates of malaria, anemia, and underweight in the RECIPAL cohort.

We found an association between malarial infections that occurred before UtA Doppler and the presence of UtA notching. The magnitude of the association was stronger with higher parasite density (ie, aOR of 4.5 for microscopic infections vs aOR of 3.3 for submicroscopic infections). In our study, the mean GA at the time of notch diagnosis was 22–23 wg. Because UtA notching can remain physiological until the 26th week of gestation, we cannot exclude that some of the detected UtA notching were physiological rather than pathological. However, late persistence of UtA notching until 22–23 wg has been suggested to reflect placental alterations because it is associated with poor birth outcomes [28]. A possible explanation is that the presence of parasites in the placenta interferes with trophoblast invasion, which is essential for placental development and then for successful fetal growth [7, 27]. Although we were expecting to find higher mean UtA-PI values in women infected with malaria, as evidenced with notching, we did not find this association. However, Espinoza et al [8] showed that UtA-PI and the presence of a UtA notch between 23 and 25 wg are independent measures, independently related to the risk of preeclampsia, whose main pathophysiological mechanism is an alteration of trophoblastic invasion.

We did not find any association between UtA-PI and malaria infections in the first trimester (ie, <15 wg) specifically, although this period is key for placental development, which is complete by 20–22 wg [7, 29]. In contrast, malaria up to 22–23 wg was associated with UtA notching. Based on previous RECIPAL findings, an explanation may be that a cumulative effect of malarial infections starting from the first trimester could be more determinant than the effect of malarial infections restricted to the first trimester only [25].

Very few studies evaluated the effect of MiP on UtA Doppler, among which 2 found an association between MiP and impaired uteroplacental blood flow [12–14]. Using a comparable longitudinal approach as in RECIPAL, Griffin et al [13] found that malarial infections in early pregnancy (<20 wg) were associated with increased UtA resistance, but only in undernourished women. In our study, the low proportion of undernourished women did not allow us to explore the association between MiP and UtA Doppler measurements according to maternal nutritional status.

In the literature, submicroscopic malaria infections have been associated with an increased risk of poor birth outcomes [30, 31]. In RECIPAL, these infections were highly prevalent and were not specifically treated [21]. In this study, they were the only infections detected in one third of our population. Our analysis suggested a positive association between submicroscopic infections and the presence of a UtA notch. To our knowledge, there is no comparable result in the literature, although submicroscopic infections have been associated with an abnormal UA Doppler [15].

We did not find any evidence supporting an association between microscopic and submicroscopic infections and abnormal UA Doppler at the end of pregnancy. This finding suggests that malarial infections did not lead to subsequent changes in fetal blood flow, which is an indicator of placental dysfunction. Our findings are in contrast with previous studies, which showed an association between MiP and increased UA resistance indices [11, 14, 15]. An explanation may be that, in our study, women were screened for microscopic infections every month and were immediately treated in case of infection. This may have mitigated the potential deleterious effect of malaria on placental dysfunction.

Finally, we performed both placental biopsies and placental thick blood smears. In the whole RECIPAL cohort, very few placental infections (n = 26 of 230) were detected using biopsies, which prevented us from assessing the association between abnormal UtA or UA Doppler with placental infection.

We acknowledge some limitations to our study. First, the proportion of primigravid women was low compared to what is generally observed in other cohorts in Africa (7.5% vs 15% to 20%) [32]. The observed differences are due to the fact that another study with a similar preconceptional design was concomitantly carried out in the same area. Primigravidae are the most susceptible to malaria, preeclampsia, and FGR [33, 34]. This may have led to an underestimation of the reported associations we found between malaria and both UtA and UA measurements. Second, we were not able to adjust for maternal hypertensive disorders, which complicate up to 10% of pregnancies worldwide and have been associated with placental vascularization impairment [35]. However, only 2.8% of RECIPAL women had gestational hypertension (none of them had a preeclampsia event). In addition, this variable was considered as an intermediate rather than a confounding factor for the association between Doppler measurements and malaria. Third, we cannot exclude that infections with ultra-low parasitemia (ie, below the PCR detection threshold) may have been missed, although we do not know their impact on Doppler measurements. Fourth, the exclusion of poor-quality Doppler images may have led to a lack of power for UtA analyses, in particular for the analysis on UtA-PI. However, a comparison of women with noninterpretable UtA Doppler and our analysis sample showed no evidence of difference. Finally, because UtA notching up to 26 wg can be of a physiological nature, we cannot exclude that some of the notches we evidenced at 22–23 wg were physiological.

## CONCLUSIONS

Despite global efforts to fight malaria, most women in SSA remain insufficiently protected against malaria during the first half of their pregnancy. Results of this study suggest that both microscopic and submicroscopic treated malaria infections that occurred in the first half of pregnancy may alter placentation. These findings highlight the importance of implementing preventive measures against malaria as early as possible during pregnancy starting in the first trimester.

## Supplementary Material

ofad376_Supplementary_Data

## Supplementary Data

Supplementary materials are available at *Open Forum Infectious Diseases* online. Consisting of data provided by the authors to benefit the reader, the posted materials are not copyedited and are the sole responsibility of the authors, so questions or comments should be addressed to the corresponding author.

## Notes

***Acknowledgments.*** We thank all the local communities of Sô-Ava and Akassato in Benin who took part in this study. We also thank all REtard de Croissance Intra-uterin et PALudisme (RECIPAL) project technical team members, midwives, nurses, and community health workers for their hard work recruiting and following the study participants. Finally, we thank Vassilis Tsatsaris (UMR1153, EPOPé research team, Paris, France) for reviewing the Doppler pictures, as well as François Nosten and Dr. Aye Min (Shoklo Malaria Research Unit, Mahidol Oxford Tropical Medicine Research Unit, Bangkok, Thailand) for their valuable comments on the analysis and findings.

***Author contributions*.** VB and GC contributed to conceptualization; WA, OTL, MM, AD, BV, and DS contributed to investigation; WA, OTL, MM, AD, BV, and DS contributed to supervision; EY, MA, CH, GA, NTN, AMa, and NF contributed to statistical analysis; AuM contributed to data interpretation; AuM, VB, GC, RM, and MJR contributed to data; AuM, VB, and GC contributed to roles/writing and original draft; AuM, EY, WA, OTL, MM, AD, MA, CH, GA, BV, DS, NTN, AMa, VB, GC, NF, RM, and MJR contributed to writing, review, and editing.

***Financial support*.** This work was supported by the Agence Nationale de la Recherche (Grant Number ANR-13-JSV1-0004) and the Fondation Simone Beer under the auspices of the Fondation de France (Grant Number 00074147).
