# Supplementary material for: Malaria Infections and Placental Blood Flow: A Doppler Ultrasound Study From a Preconception Cohort in Benin
Source: Open Forum Infect Dis. 2023 Aug 10;10(8):ofad376. doi: 10.1093/ofid/ofad376 (PMC10414806; doi:10.1093/ofid/ofad376)
Supplement: ofad376_Supplementary_Data [file ofad376_supplementary_data.zip › Mondeilh_Malaria-Doppler_Supplementary_Tables_final.docx]

**Supplementary table 1**: Comparison of general characteristics between women excluded (n=158) and included (n=253) in the analyses, RECIPAL cohort, southern Benin, 2014-2017.

| **Characteristics** | **Excluded women (n=158)** | |  | **Included women (n=253)** | |
| --- | --- | --- | --- | --- | --- |
|  | **Total** | **N (%)  or mean (SD)** |  | **Total** | **N (%)  or mean (SD)** |
| Maternal age (years) | 158 | 26.8 (5.1) |  | 253 | 26.7 (5.0) |
| Gravidity | 158 |  |  | 253 |  |
| Primigravidae |  | 14 (8.9) |  |  | 19 (7.5) |
| Secondigravidae |  | 34 (21.5) |  |  | 32 (12.6) |
| Multigravidae |  | 110 (69.6) |  |  | 202 (79.8) |
| Education level (illiterate vs literate) | 158 | 109 (69.0) |  | 253 | 181 (71.5) |
| Pre-pregnancy body mass index (kg/m^2^) | 158 |  |  | 253 |  |
| < 18.5 |  | 17 (10.8) |  |  | 22 (8.7) |
| 18.5 - 24 |  | 93 (58.9) |  |  | 171 (67.6) |
| ≥ 25 |  | 48 (30.4) |  |  | 60 (23.7) |
| Socioeconomic status^a^ | 158 |  |  | 253 |  |
| Low tercile |  | 53 (33.5) |  |  | 87 (34.4) |
| Middle tercile |  | 69 (43.7) |  |  | 98 (38.7) |
| High tercile |  | 36 (22.8) |  |  | 68 (26.9) |
| HIV status (positive vs negative) | 108 | 2 (1.9) |  | 246 | 4 (1.6) |
| Anemia before conception (≤ 12 g/dl)^b^ | 155 | 75 (48.4) |  | 253 | 146 (57.7) |
| Ethnic group (Toffin vs others^c^) | 158 | 113 (71.5) |  | 253 | 187 (73.9) |
| Previous miscarriages (yes *vs.* no) | 158 | 56 (35.4) |  | 253 | 99 (39.1) |
| Previous stillbirth (yes *vs.* no) | 158 | 8 (5.1) |  | 253 | 13 (5.1) |
| Previous preterm birth (yes *vs.* no) | 142 | 5 (3.2) |  | 253 | 10 (4.0) |
| Previous Gestational hypertension (≥ 1 episode *vs.* no episode) | 142 | 5 (3.2) |  | 253 | 6 (2.4) |

Abbreviations: REtard de Croissance Intra-uterin et PALudisme (RECIPAL) ; Standard Deviation (SD); Human Immunodeficiency Virus (HIV).

^a^ Socioeconomic status: synthetic score combining occupation and ownership of assets, which was then categorized according to the tertiles (24).

^b^Anaemia detected at recruitment in the preconception period, in median 12.3 months before pregnancy (17).

^c^ Other ethnic groups: Fon, Aïzo, Yoruba, Adja, Goun, Ahoussa, Cotafon, Mahi, and Sahoue.

**Supplementary table 2**: Comparison of general characteristics and exposure to malaria between women included in (n=175) and excluded from (n=69, due to non-interpretable UtA Doppler) the Uterine Artery (UtA) Doppler analyses, RECIPAL cohort, southern Benin, 2014-2017.

| **Characteristics** | **Excluded women (n=69)** | | **Included women (n=175)** | |
| --- | --- | --- | --- | --- |
|  | **Total** | **N (%)  or mean (SD)** | **Total** | **N (%)  or mean (SD)** |
| Maternal age (years) | 69 | 25.9 (4.4) | 175 | 27.0 (5.1) |
| Gravidity | 69 |  | 175 |  |
| Primigravidae |  | 6 (8.7) |  | 13 (7.4) |
| Secondigravidae |  | 10 (14.5) |  | 21 (12.0) |
| Multigravidae |  | 53 (76.8) |  | 141 (80.6) |
| Education level (illiterate vs literate) | 69 | 50 (72.5) | 175 | 124 (70.9) |
| Pre-pregnancy body mass index (kg/m^2^) | 69 |  | 175 |  |
| < 18.5 |  | 4 (5.8) |  | 17 (9.7) |
| 18.5 - 24 |  | 51 (73.9) |  | 114 (65.1) |
| ≥ 25 |  | 14 (20.3) |  | 44 (25.1) |
| Socioeconomic status^a^ | 69 |  | 175 |  |
| Low tercile |  | 26 (37.7) |  | 58 (33.1) |
| Middle tercile |  | 28 (40.6) |  | 68 (38.9) |
| High tercile |  | 15 (21.7) |  | 49 (28.0) |
| HIV status (positive vs negative) | 66 | 1 (1.4) | 171 | 3 (1.7) |
| Anemia before conception (≤ 12 g/dl)^b^ | 69 | 38 (55.1) | 175 | 104 (59.4) |
| Ethnic group (Toffin vs others^c^) | 69 | 59 (85.5) | 175 | 122 (69.7) |
| Malaria before or at time of UtA Doppler | 69 |  | 175 |  |
| No infection |  | 24 (34.8) |  | 70 (40.0) |
| Submicroscopic infection(s) only ^d^ |  | 22 (31.9) |  | 56 (32.0) |
| At least one microscopic infection ^e^ |  | 23 (33.3) |  | 49 (28.0) |

Abbreviations: REtard de Croissance Intra-uterin et PALudisme (RECIPAL); Uterine Artery (UtA); Standard Deviation (SD); Human Immunodeficiency Virus (HIV).

^a^Anaemia detected at recruitment in the preconception period, in median 12.3 months before pregnancy (17).

^b^ Socioeconomic status: synthetic score combining occupation and ownership of assets, which was then categorized according to the tertiles (24).

^c^ Other ethnic groups: Fon, Aïzo, Yoruba, Adja, Goun, Ahoussa, Cotafon, Mahi, and Sahoue.

^d^ At least one submicroscopic infection and no microscopic infection before the Doppler measurement.
^e^ At least one microscopic infection before the Doppler measurement.

**Supplementary table 3:** Crude association between Uterine Artery (UtA) *notching* and poor birth outcomes (n=175). RECIPAL cohort, southern Benin, 2014-2017.

| **Characteristics** | **n** | **Absence of a *notch^a^***  **n (%)** | **Presence of a *notch^a^***  **n (%)** | **p-value*** |
| --- | --- | --- | --- | --- |
|  |  |  |  |  |
| SGA^b^ |  |  |  | 0.51 |
| No | 137 | 117 (85.4) | 20 (14.6) |  |
| Yes | 35 | 32 (91.4) | 3 (8.6) |  |
| Low birth weight (<2500 g) |  |  |  | 0.82 |
| No | 158 | 137 (86.7) | 21 (13.3) |  |
| Yes | 16 | 13 (81.2) | 3 (18.8) |  |
| Preterm birth |  |  |  | 0.15 |
| No | 161 | 141 (87.6) | 20 (12.4) |  |
| Yes | 13 | 9 (69.2) | 4 (30.8) |  |

Abbreviations: REtard de Croissance Intra-uterin et PALudisme (RECIPAL); Uterine Artery (UtA); Small-for-Gestational Age (SGA).

* Chi squared test (with Yates correction)

^a^ Presence on the right or left uterine arteries.

^b^ Defined as birthweight below the 10^th^ centile for a given gestational age (GA) according to INTERGROWTH 21st’s charts (17).

**Supplementary table 4:** Crude association between abnormal Umbilical Artery (UA) doppler and poor birth outcomes (n=250). RECIPAL cohort, southern Benin, 2014-2017.

| **Characteristics** | **n** | Normal UA Doppler n (%) | Abnormal UA Doppler ^a^ n (%) | **p-value*** |
| --- | --- | --- | --- | --- |
|  |  |  |  |  |
| SGA^b^ |  |  |  | 0.02 |
| No | 196 | 175 (89.3) | 21 (10.7) |  |
| Yes | 51 | 39 (76.5) | 12 (23.5) |  |
| Low birth weight (<2500 g) |  |  |  | 0.54 |
| No | 226 | 195 (86.3) | 31 (13.7) |  |
| Yes | 22 | 20 (90.9) | 2 (9.1) |  |
| Preterm birth |  |  |  | 0.92 |
| No | 229 | 198 (86.5) | 31 (13.5) |  |
| Yes | 21 | 18 (85.7) | 3 (14.3) |  |

Abbreviations: REtard de Croissance Intra-uterin et PALudisme (RECIPAL); Umbilical Artery (UA); Small for Gestational Age (SGA).

* Chi squared test

^a^ Defined as: Z-score ≥ 2 Standard Deviation (equivalent to UA pulsatility index ≥ 98^th^ percentile) or absent/reversed UA end-diastolic flow.

^b^ Defined as birth weight below the 10^th^ centile for a given gestational age (GA) according to INTERGROWTH 21st’s charts (17).

**Supplementary figure n° 1**: Study design, RECIPAL cohort, southern Benin, 2014-2017

Abbreviations: Antenatal care visit (ANC visit); Thick blood smear (TBS); Polymerase chain reaction (PCR); Rapid Diagnostic Test (RDT); weeks of gestation (wg); REtard de Croissance Intra-uterin et PALudisme (RECIPAL).
