# Supplementary figures and images for: Malaria Infections and Placental Blood Flow: A Doppler Ultrasound Study From a Preconception Cohort in Benin
Source: Open Forum Infect Dis. 2023 Aug 10;10(8):ofad376. doi: 10.1093/ofid/ofad376 (PMC10414806; doi:10.1093/ofid/ofad376)

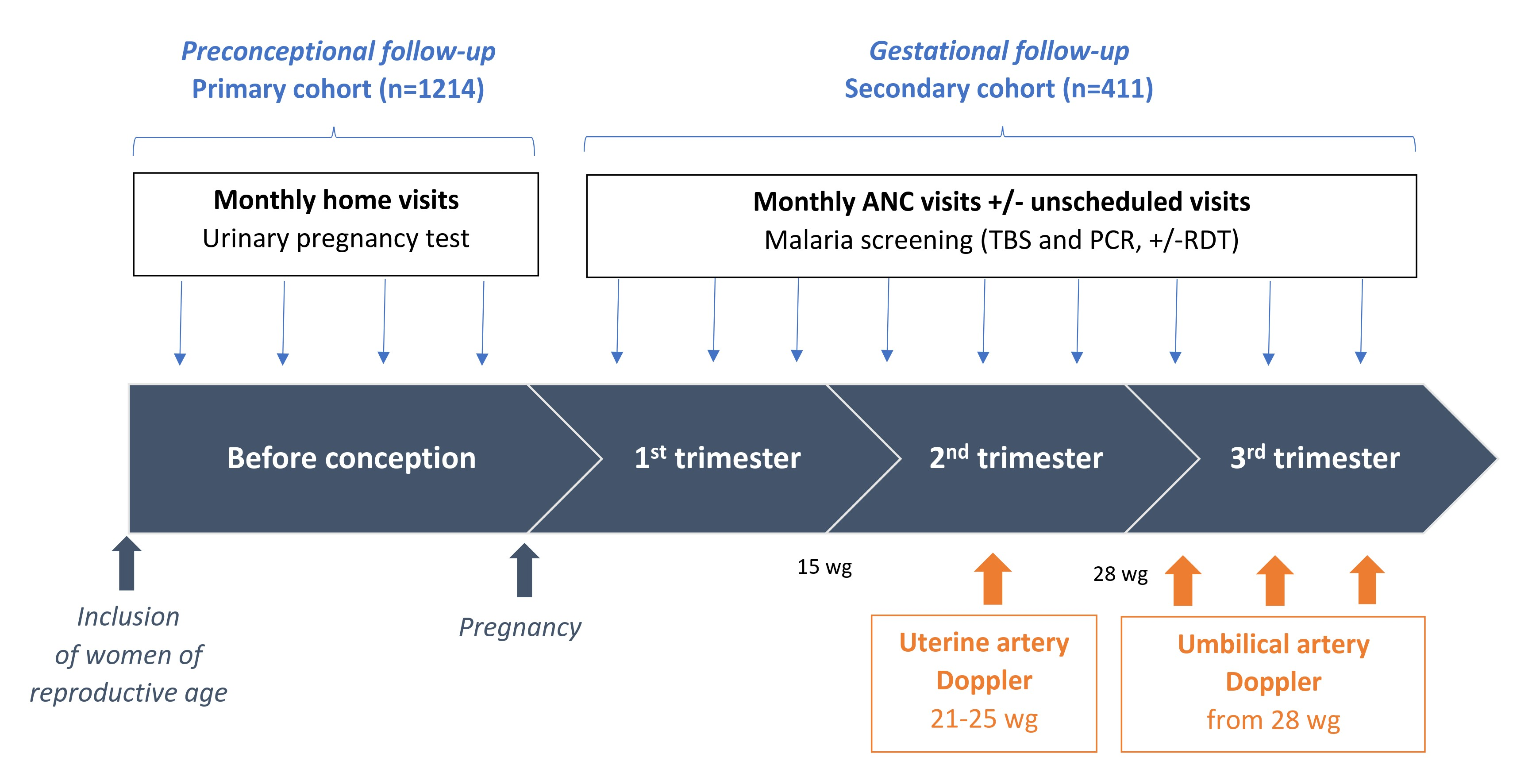

Supplement: ofad376_Supplementary_Data [file ofad376_supplementary_data.zip › Supplementary_figure_1_design.png]
